# Time-Course of Motor Involvement in Literal and Metaphoric Action Sentence Processing: A TMS Study

**DOI:** 10.3389/fpsyg.2019.00371

**Published:** 2019-02-26

**Authors:** Megan Reilly, Olivia Howerton, Rutvik H. Desai

**Affiliations:** ^1^Department of Psychology, University of South Carolina, Columbia, SC, United States; ^2^Institute for Mind and Brain, University of South Carolina, Columbia, SC, United States

**Keywords:** embodied cognition, grounded cognition, metaphor semantics, abstract semantics, non-invasive brain stimulation, transcranial magnetic stimulation

## Abstract

There is evidence that the motor cortex is involved in reading sentences containing an action verb (“The spike was hammered into the ground”) as well as metaphoric sentences (“The army was hammered in the battle”). Verbs such as ‘hammered’ may be homonyms, with separate meanings belonging to the literal action and metaphoric action, or they may be polysemous, with the metaphoric sense grounded in the literal sense. We investigated the time course of the effects of single-pulse transcranial magnetic stimulation to primary motor cortex on literal and metaphoric sentence comprehension. Stimulation 300 ms post-verb presentation impaired comprehension of both literal and metaphoric sentences, supporting a causal role of sensory-motor areas in comprehension. Results suggest that the literal meaning of an action verb remains activated during metaphor comprehension, even after the temporal window of homonym disambiguation. This suggests that such verbs are polysemous, and both senses are related and grounded in motor cortex.

## Introduction

Multiple lines of evidence support the view that concepts are grounded at least partially in sensory-motor systems of the brain. This view falls under the umbrella of grounded or embodied cognition, the notion that cognition is inseparably linked with perceptual systems (Barsalou, 1999; Gallese and Lakoff, 2005; Pulvermüller, 2013). In the last two decades, a body of behavioral, neuroimaging, electrophysiological, and patient-based studies have supported this view (Fischer and Zwaan, 2008; Binder and Desai, 2011; Kiefer and Pulvermuller, 2012; Meteyard et al., 2012). This is in contrast to the traditional view (e.g., Caramazza et al., 2014; Mahon, 2015) which suggests that concepts are abstract and symbolic, and sensory-motor information does not play a significant role in their representation. On this view, brain activations and behavioral effects seen in studies play no role or a relatively minor role in concept representation, for example as an embellishment to the concept. They are posited to be epiphenomenal as opposed to essential, and represent spreading activation after the primary task of comprehension has taken place through amodal mechanisms. Hybrid proposals also exist, where sensory-motor systems are involved in concept representation, but only under certain circumstances within a flexible, multi-level system, and are not always necessary (Kemmerer, 2015).

Studies of patients with sensory or motor impairments constitute stronger evidence for conceptual embodiment, because they speak directly to the causal involvement of sensory-motor systems in concept representation and processing. For example, many examples of motor-related linguistic deficits have been reported in patients with Parkinson’s Disease (PD), suggesting that motor system degeneration in PD causes a deficit in processing actions. Findings have included processing actions relative to objects (Cotelli et al., 2006; Boulenger et al., 2008); action verbs relative to abstract verbs (Fernandino et al., 2013a); and literal and figurative motion relative to abstract verb sentences (Fernandino et al., 2013b), although not all PD studies show accuracy deficits for action verbs (Kemmerer et al., 2013). These effects emerge even in the absence of a general mild cognitive impairment (Bocanegra et al., 2015, 2017) and are correlated with patients’ sensitivity to the action-sentence compatibility effect (ACE) in which a participant’s physical response is facilitated if the movement is congruent with the action taking place in a probe sentence (Ibáñez et al., 2013). Similarly, patients with amyotrophic lateral sclerosis (ALS) are more impaired for action knowledge than for object knowledge (Grossman et al., 2008). In chronic stroke patients, processing deficits for action words was predicted by their degree of impairment in a reaching task (Desai et al., 2015). Patient evidence for embodiment is not limited to actions and the motor system; for example, in a case study Trumpp et al. (2013) described a patient with damage to auditory association cortex who had specific impairments for sound-related conceptual information.

Transcranial magnetic stimulation (TMS) also provides a valuable tool for addressing the question of brain-behavior causality. TMS uses a non-invasive focal magnetic pulse to disrupt the activity of the underlying cortex. Single-pulse TMS also allows for precise control over the timing of stimulation. The effects of a single pulse of TMS last up to a few seconds, allowing an experimenter to manipulate stimulation at the level of a single trial. It should be noted, however, that TMS has downstream effects, such that targeting a single region may also impact areas to which it is functionally connected (Hauk and Tschentscher, 2013). Pulvermüller et al. (2005) showed that lexical decisions made on action verbs are selectively affected by TMS to the subregion of the primary motor cortex involved in performing that action. For example, TMS to the hand area of the motor cortex affects recognition of hand-related words (e.g., ‘fold’) relative to leg-related words (‘kick’), and the opposite results emerged for TMS to the leg area. Similarly, Buccino et al. (2005) showed that if participants are listening to hand-action sentences, motor-evoked potentials (MEPs) in the hand muscle are selectively reduced in response to hand motor cortex TMS. The same effect emerged for leg-action sentences and TMS to the leg motor cortex. TMS to the hand motor cortex has also been shown to modulate the ACE (Glenberg et al., 2008). These results not only suggest that the primary motor cortex plays a causal role in action word recognition, but that this role in language processing follows the same somatotopic map as performing those actions. It has recently been suggested that the effects of motor cortex TMS on language is task-specific, such that an explicit semantic task elicits an effect but semantically shallow tasks such as lexical decision do not (Vukovic et al., 2017).

Metaphors represent an interesting case for concept grounding, because of their similarity to both concrete and abstract domains. Several imaging studies have examined the question of how metaphors are represented in the brain, and whether the comprehension of a metaphor is grounded in the neural regions associated with the literal meaning of that word. For example, multiple studies (Desai et al., 2011, 2013) have shown that action verbs activate a higher-order sensory-motor region when they are used literally *or* metaphorically, suggesting that the metaphoric sense is indeed based on the literal sense (Aziz-Zadeh et al., 2006; Boulenger et al., 2009; Lacey et al., 2012). One TMS study to our knowledge has used TMS to investigate the grounding of metaphors in the motor cortex. Cacciari et al. (2011) applied TMS to the primary leg motor area and examined MEPs in the leg muscles during comprehension of literal, metaphorical, idiom or fictive motion (“The road turned left”) sentences. TMS to the leg muscle reduced MEPs during literal leg-action sentences, similar to the results of Buccino et al. (2005). Of interest, they also found that metaphors showed a similar effect to literal sentences, suggesting that metaphors are obligatorily processed in the primary motor cortex. However, the authors drew their conclusions based on a null difference between the metaphor and literal sentences, not based on a difference from a baseline condition. Additionally, the effects of motor cortex TMS on psycholinguistic behavior remain unclear. The current study aims to examine the causal role of motor cortex involvement during both metaphoric and literal action sentence processing using TMS, as well as the time course of this involvement.

If the motor cortex maintains access to literal action verb meanings during metaphor comprehension, how long does this activity last? If TMS is applied after the literal meaning of an action verb is no longer available, the stimulation will have no effect on sentence comprehension. Thus, the stimulus onset asynchrony (SOA) of TMS can be manipulated to examine how late in sentence processing the motor cortex is playing a role. Here, we might view metaphor comprehension in terms of comprehending an ambiguous word. Many ambiguous words, such as “pen,” have both a dominant or more frequent meaning (the writing implement) and a subordinate meaning (an animal cage). When the subordinate meaning is intended, words with strong biases receive longer gaze durations than words with more balanced meanings (Duffy et al., 1988; Rayner et al., 1994). The predictability and relatedness of the sentence context also has a substantial influence on disambiguation (Schwanenflugel and Shoben, 1983; Schwanenflugel and White, 1991; Binder, 2003). These findings have been reconciled in “ordered access” models, in which accessibility of an ambiguous word’s meanings is weighted by both the dominance of the meaning and the information provided by context (Morris, 2006). Importantly, these models assume that all possible meanings are initially accessed. This research attempts to explain the time course of disambiguation in terms of how long it takes for the intended meaning to be selected successfully. The current study investigates how long the putatively incongruent meaning remains accessible: specifically, how long the literal meaning of an action verb remains accessible during metaphor comprehension, if it is activated at all.

One possibility is that the literal interpretation of a verb is suppressed as soon as the metaphor is detected. This hypothesis assumes that the literal and metaphor meanings are interpreted as homophones, with two competing and mutually exclusive meanings. This is consistent with Indirect Access models of metaphors, which posit that the metaphorical meaning of a concept necessarily follows access to (and rejection of) its literal interpretation (Searle, 1979; Janus and Bever, 1985). An alternative view argues for direct access to the metaphoric meaning without activation of the literal interpretation (Gibbs, 1994; Glucksberg, 2008). Behavioral, ERP, and eye-tracking evidence suggests that in homophones, the congruent meaning of an ambiguous word is resolved very quickly following word presentation (Simpson, 1981; Swinney, 1979; Tanenhaus and Leiman, 1979; Onifer and Swinney, 1981; Sereno et al., 2003; Sheridan et al., 2009). For example, Seidenberg et al. (1982) found that early in processing, recognition of an ambiguous word (e.g., “straw”) can be primed by activation of either meaning (“sip”/“hay”), even if its sentence context requires a single meaning (“The farmer bought the straw”). However, if the prime is presented 200 ms after the sentence, the priming effect only exists for congruent primes (“hay”), suggesting that the incongruent meaning of “straw” is no longer available after 200 ms. In an analysis of first fixation durations, Sheridan and Reingold (2012) found that dominant and subordinate meanings of ambiguous words can be distinguished by as early as 139 ms. Peak EEG signal differences between subcategories of action words (e.g., arm and leg) are detected in motor areas around 220 ms (Hauk and Pulvermuller, 2004). From a neural perspective, if the literal meaning of an action verb does not play a causal role in processing metaphors, then motor cortex involvement in processing should also cease approximately within the first 200 ms. Thus, the homophone view of metaphors predicts that motor cortex involvement in metaphor comprehension should *not* emerge if TMS occurs later than 200–220 ms after verb presentation, after suppression of the literal meaning is complete.

An alternative view of metaphors postulates that metaphor meanings are polysemous with their literal counterparts (Apresjan, 1974). By this view, literal and figurative meanings are related (e.g., grasp a handle vs. grasp an idea), and their neural representations are inextricably linked. Activation of one sense facilitates the other, a phenomenon known as the ambiguity advantage: lexical decisions for homophones tend to be slower than for unambiguous words, but for polysemous pairs RTs tend to be faster (Borowsky and Masson, 1996; Hargreaves et al., 2011). Eye-tracking evidence supports the view that the semantic system does not make a “commitment” to a single interpretation of an ambiguous word unless its possible meanings are not mutually compatible (Frazier and Rayner, 1990). Magnetoencephalography (MEG) suggests that homophonous and polysemous ambiguity are neurally separable as well (Beretta et al., 2005); both behavioral RTs and the M350 MEG response were earlier for polysemous words than unambiguous words, and slower for homophones.

Thus, the homonymy view of metaphor comprehension suggests that the unrelated literal meaning is briefly activated and then suppressed (or not activated at all), and does not play a causal role in metaphor comprehension. The polysemy view suggests that the metaphoric and literal senses are related, hence the literal sense is sustained even late in metaphor comprehension.

In the current study, access to the literal meaning is likely to involve the motor cortex. ERP measurements have shown topographically organized motor strip activity during action verb processing as early as 250 ms; e.g., selective activity for hand-action verbs in the hand-motor cortex (Pulvermuller et al., 2001). According to embodied theories of cognition, motor cortex activation while processing action metaphors should be relatively sustained and causally related to comprehension, because the metaphoric sense is grounded in the literal sense.

The ERP studies of metaphors have frequently reported a biphasic pattern, with a centro-parietal negativity (N400) and a later parietal positivity (P600) (e.g., Coulson and Van Petten, 2002; De Grauwe et al., 2010; Weiland et al., 2014; Schmidt-Snoek et al., 2015; Bambini et al., 2016). Studies finding a later effect (P600) tend to favor the indirect access model, while those finding an N400 component argue for a direct access view. These studies do not directly address the question of how the metaphoric meaning is related to the literal meaning, and whether a metaphoric action can be grounded in the literal sense, even if accessed directly. While ERP studies have not yet resolved the direct vs. indirect access debate, if P600 is interpreted as a reanalysis stage (Yang et al., 2013), it can be viewed as involving discarding of the initial literal meaning and re-interpretation with a separate homonymous abstract meaning.

Here, we use single-pulse TMS on hand motor cortex at three stimulus onset asynchronies (SOAs) post-verb presentation (150, 300, and 450 ms) to investigate the timing of the role of the motor cortex in processing literal action sentences and action metaphors. The short (150 ms) SOA acts as a control timepoint, at which TMS is not expected to strongly impact semantic processing, as lower-level processes such as orthographic processing dominate during this time window. At the 300 ms SOA, the first question addressed here is whether the motor cortex has a causal role in metaphoric or literal action sentence processing. If the activation seen in imaging studies is epiphenomenal, no effects of TMS are expected relative to control stimulation, while a causal role of motor cortex predicts modulation in RT. Secondly, the homophony and polysemy theories diverge at 300 ms. Homophone word meanings are expected to be resolved well before 300 ms, and the homophone view predicts that the incongruent literal meaning of the action verb should be suppressed during metaphor processing. No effects of motor cortex TMS should be observed at 300 ms for action metaphors. The polysemy view, on the other hand, suggests a sustained activation of action meaning, and hence predicts an effect of TMS at 300 ms for metaphoric sentences. Specifically, we hypothesized that at 300 ms, both literal and metaphoric RTs will be modulated relative to control stimulation. The 450 ms timepoint tests further sustainment of action meaning, although the influence of verb meaning may be reduced at this point as subsequent words are also available for processing. We collected RTs in a meaningfulness judgment task as the primary measure of interest. We also recorded MEPs as an additional, secondary measure, although the design was not ideal for measuring MEPs due to the interference caused by the manual response.

## Materials and Methods

### Participants

Thirty-three participants were recruited from the subject pool at the University of South Carolina. Participants were screened such that they fit all of the following criteria: right-handed according to the Edinburgh Handedness Inventory, native English speaker, and are at minimal risk for a seizure (excluding potential participants who: have epilepsy or family history of epilepsy; have a history of fainting, head trauma, head injury or concussion; have potentially magnetic implants; or are taking a prescription or over-the-counter medication which might lower seizure threshold). Participants provided informed written consent and received either course credit or a stipend for their participation. The methods used in this study were approved in advance by the Institutional Review Board at the University of South Carolina. All human subjects research was performed in compliance with the IRB.

Of the participants who participated, 9 failed to respond with at least 90% accuracy. Because there were relatively few stimuli in each condition and inaccurate responses were removed, these 9 participants were excluded from the analysis to maintain a more reliable estimate per participant. Of the remaining 24 participants whose data are reported here (15 female, average age 19.48), mean accuracy was 97.2% (SD 1.65%).

### Motor Thresholding and MEP Measurements

Prior to the experimental procedure, the resting motor threshold for each participant was determined. Electrodes were placed on the participant’s first dorsal interosseus (FDI) muscle. The hand motor cortex was localized by determining the location on the scalp that elicited the strongest average motor-evoked potential (MEP), initially estimated using the hotspot 5 cm lateral to the vertex. The amplitude for stimulation was then thresholded using incremental adjustments until a stimulation level was determined that elicited a noticeable MEP spike (at least 50 μV) or visible muscle twitch in response to 5 out of 10 consecutive stimulations. This amplitude was identified as that participant’s resting motor threshold (RMT).

### Stimuli and Procedure

Stimuli were the sentences used by Desai et al. (2013). Sentences belonged to one of three conditions: literal action (“The craftsman lifted the pebble from the ground.”), metaphoric action (“The discovery lifted the nation out of poverty”), or abstract (“The country wanted the plan for a nuclear program”). The literal and metaphoric sentences contained the same hand/arm action verb, while the abstract sentence contained a verb that was unlikely to be associated with a physical action, as normed by Desai et al. (2013). Metaphoric and abstract sentences contained an abstract or collective head noun (e.g., *the discovery*, *the university*) making a literal interpretation of the verb unlikely or impossible. The literal sentences contained an animate agent (e.g., *the craftsman*, *the doctor*). Thus, when the verb is encountered, literal sentences allow for both concrete and abstract/metaphoric interpretation of the verb, while metaphoric and abstract sentences only allow for the abstract/metaphoric interpretation of the verb. These noun phrases provided the context in which the verbs were interpreted. Literal, metaphor, and abstract sentences were normed by a separate group of 20 participants in Desai et al. (2013). Norming participants judged whether each sentence was meaningful; there were no pairwise differences between conditions on the reaction times during this task [mean (SD) reaction times were: literal, 1628 (343); metaphor, 1649 (358), and abstract, 1596 (356)]. Thus, it is unlikely that the literal meanings of the action verbs were interpreted as significantly more dominant than the metaphor meanings. In addition to the 40 sentences in each condition, 52 filler sentences were used which either contained a novel verb (e.g., “He learned a new skill”) or had content words replaced with nonsense (e.g., “All the dom occeniow more lecese”). Further details about preparation and norming of the stimulus materials are reported in Desai et al. (2013).

Sentences were presented visually in two parts, matching the presentation in the imaging study: the subject noun phrase was presented for 500 ms, followed by the remainder of the sentence, beginning with the critical verb (“The discovery/lifted the nation out of poverty”). Following the removal of one low-familiarity sentence as described below, the length (in characters) of the initial noun phrase was slightly longer for Literal ((16.1) than for Metaphor (13.8) or Abstract (13.9) sentences, which differed significantly [*F*(2,110) = 3.256, *p* = 0.0423]. However, the portion of the stimulus containing the critical verb and triggered a response did not differ (Literal: 27.7, Metaphor: 29.3, Abstract: 27.8), nor were there any significant differences between SOAs nor interactions between Condition and SOA. As reported in Desai et al. (2013), the abstract nature of those sentences did not allow for perfectly balanced frequency of the main verb. The log SUBTL frequency for the main verb (Brysbaert and New, 2009) was higher on average for Abstract sentences (3.17) than for Literal (2.74) or Metaphor (2.76) sentences, although again the average frequencies did not differ between SOAs. Finally, an ANOVA on the semantic neighborhood density of critical verbs (Reilly and Desai, 2017) did not reveal any differences between SOAs or Conditions, nor an interaction.

Participants were asked to indicate by button press whether each sentence made sense (sentences containing nonsense words were used as a control; all sentences in experimental conditions elicited ‘yes’ responses). All participants responded with their left hands; electrodes were attached to the right FDI in order to monitor MEPs throughout the experiment. Participants were instructed to relax their right hands as much as possible throughout the task.

A total of 86 sentences were presented for each stimulation site (see section “Stimulation Protocol” below), divided into a total of four experimental runs of approximately 6 min each. Each sentence was randomly assigned to one of three stimulus onset asynchronies (150, 300, and 450), and sentence presentation was counterbalanced across participants such that each sentence appeared during motor cortex stimulation for half of participants and during control site stimulation for the other half.

### Familiarity and Conditional Probability Measurements

Previous findings suggest that metaphors tend to be less familiar overall than literal sentences or idioms (Desai et al., 2013; Romero Lauro et al., 2013). Thus, the familiarity of each sentence was measured by ratings collected using Amazon Mechanical Turk. Each sentence was scored by 7 raters, who were asked to rate each sentence on a scale of 1–7, 1 being extremely unfamiliar and 7 being extremely familiar. Nonsense sentences were included to validate the results, and all participants rated these filler sentences with very low familiarity. One literal sentence was removed from the analysis for having a particularly low score (over 3 standard deviations below the mean for non-filler sentences). No significant differences in familiarity were found (using a Wilcoxon Rank Sum Test for each pair of conditions in R, familiarity ∼ condition; all *p* > 0.10). Mean (SD) familiarity scores were 5.87 (0.66) for Abstract, 5.85 (0.65) for Literal, and 5.57 (0.84) for Metaphor.

In order to ensure that sentence contexts did not differ in the subject-verb predictability between literal and metaphor sentences, conditional probabilities for each subject-verb pair in the literal and metaphor conditions were calculated using the Web Language Model from Microsoft Cognitive Services^1^. If a verb is used predominantly in a literal or metaphoric sense, one would expect the corresponding conditional probabilities to be higher. A *t*-test (log conditional probability ∼ condition, in R) within each verb showed no significant difference between literal (mean -6.00, *SD* 1.22) and metaphor (mean -6.22, *SD* 0.96) log conditional probabilities [*t*(19) = 0.98, *p* > 0.1]. The same analysis was performed using the entire noun phrase (“The repairman” – “bent”), which also showed no difference between metaphor [-5.954 (*SD* 1.08)] and literal [-5.616 (*SD* 1.37)] conditions [*t*(36) = -0.87, *p* > 0.1].

### Stimulation Protocol

**FIGURE 1:**
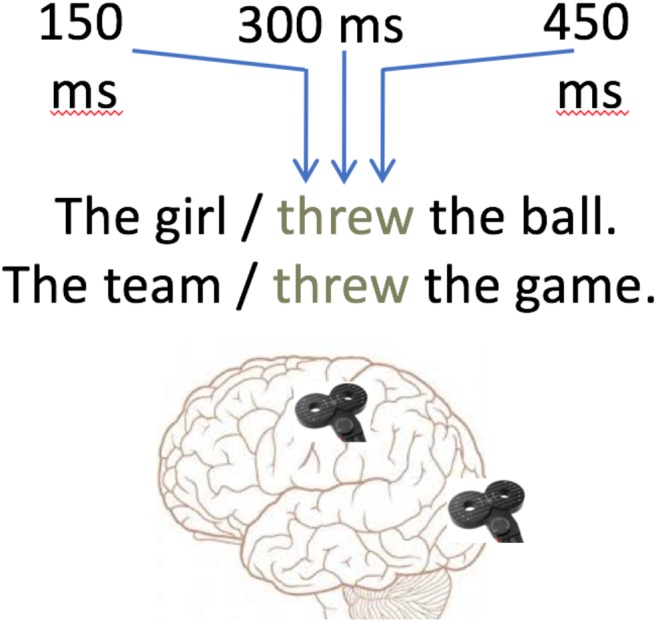
Example trial sentences and stimulation sites.

Single-pulse TMS was applied using a MagVenture MagPro stimulator and a figure-of-eight coil. Two sites of stimulation were used in two within-subject blocks in randomized order: the left primary hand cortex (Motor Site) and the occipital pole (Control Site) 2 cm superior to the inion along the vertex (Figure 1). A single biphasic pulse was applied during every trial at one of three SOAs following verb presentation: 150, 300, and 450 ms. Inter-trial intervals ensured at least 5 s between pulses. In order to avoid stimulation of perceptual or language areas during the control site stimulation, control site stimulation was performed at 90% of the RMT. The use of 90% threshold control stimulation was selected to resemble a sham TMS condition (Lisanby et al., 2001; Rossi et al., 2009), but to induce the discomfort associated with real TMS in the experimental condition. The lower threshold was used to avoid the disrupting effects of phosphenes in the visual field (Kammer et al., 2001), in order to prevent behavioral effects due to disruption in reading rather than due to semantics or general effects of TMS. No participants in the current study reported seeing phosphenes, suggesting that the stimulation was not sufficient to cause impairment in visual processing. TMS at the motor hotspot was at 110% of the RMT. Two participants reported an occasional peripheral blink or face twitch during the motor cortex stimulation, but overall these effects were minimal.

### Statistical Analysis

Reaction times (RTs), measured from the presentation of the verb, were analyzed only for correct responses (the average participant’s accuracy rate was 97.2% of stimuli). For both RT and MEP analyses, results were *z*-scored within-participant. Trials were removed if RTs were more than 2.5 standard deviations above or below the mean RT for each participant in each condition. This removed 0.2% of the data. Following this transformation, the low-familiarity sentence was removed from the analysis. Note that because sentences were randomly assigned to an SOA and one Literal sentence was removed from the analysis for being too unfamiliar, the number of sentences in a given condition and SOA varies between 12 and 14, leading to slight variability in the degrees of freedom across hypothesis tests that are performed on individual SOAs or conditions. Specifically, the number of stimuli in each SOA (150, 300, and 450, respectively) in each condition were: Abstract (13, 13, and 14); Literal (14, 12, and 13); Metaphor (13, 14, and 13).

In the RT analysis, TMS effects were measured as a difference score between RTs at the Motor and Control sites. These difference scores were subjected to analysis of variance (ANOVA) and a mixed effects model in R (R-Core-Team, 2014) in order to examine RTs while taking both Subject and Item variance into account. In the MEP analysis, similar analyses were performed using the Motor site MEPs rather than a difference score. *Post hoc* *t*-tests were used to examine differences between pairs of conditions or SOAs when omnibus main effects were present.

## Results

The mean raw RTs in each condition are listed in Table 1.

**Table 1:** Mean (SD) RTs for each condition in each site of stimulation.

Motor	Abstract	Literal	Metaphor
150 ms	2778 (192)	2791 (157)	2863 (107)
300 ms	2863 (210)	2892 (156)	2902 (135)
450 ms	2825 (205)	2935 (174)	2850 (149)
**Control**	**Abstract**	**Literal**	**Metaphor**

150 ms	2773 (208)	2745 (140)	2827 (138)
300 ms	2868 (210)	2785 (156)	2804 (203)
450 ms	2824 (217)	2843 (217)	2913 (174)


*Note that this is an item analysis; each item’s mean RT was averaged and the mean (SD) of those averages is reported here.*

The critical analysis concerns whether TMS affects the different sentence conditions differently relative to the control stimulation, and whether this effect shows an interaction with SOA. Thus, an item analysis was performed using the difference between TMS and control site RTs for each condition. For each sentence, the average reaction times were calculated within each Site, and the average RT during motor site stimulation was subtracted by the average RT during control site stimulation. Thus, the results reported here are a by-item difference score. A value of zero indicates that TMS to the motor cortex did not have any effect on RTs relative to the control site. A positive value indicates that motor cortex TMS elicits slower RTs than control site TMS. Because each sentence was presented to half of the participants during control stimulation and half during motor site TMS, also counterbalancing the order of site stimulation across participants, the difference score analysis also accounts for any repetition effects of the repeated verbs across metaphor and literal sentences.

**FIGURE 2:**
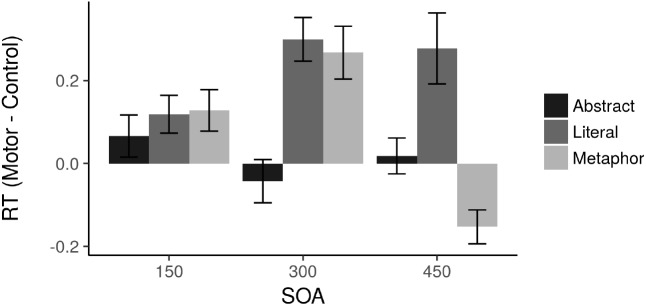
Effects of TMS on RTs. Positive values represent slower RTs following TMS.

Figure 2 depicts the RT results. A by-item omnibus ANOVA in *R* (that is, collapsing results across subjects and treating item as the random variable in a repeated measures ANOVA) showed a main effect of Condition [*F*(2,110) = 3.929, *p* = 0.023, η^2^ = 0.060] and an interaction between SOA and Condition [*F*(4,110) = 2.589, *p* = 0.040, η^2^ = 0.079] with no main effect of SOA. There was a main effect of Condition within both the 300 ms [*F*(2,36) = 3.679, *p* = 0.035, η^2^ = 0.167] and 450 ms [F(2,37) = 4.374, *p* = 0.020, η^2^ = 0.191] SOAs but not the 150 ms SOA (*F* < 1, *p* > 0.1). The abstract sentence difference scores were not significantly different from zero at any SOA, and no conditions were significantly different from zero at the 150 ms SOA. Comparing the Literal and Metaphor results to the Abstract sentences, there were no pairwise significant differences between conditions at the 150 ms SOA.

The critical hypotheses concern the 300 ms SOA. Within this SOA, both Literal [*t*(11) = 3.149, *p* = 0.009] and Metaphor [*t*(13) = 2.52, *p* = 0.026] sentences were significantly different from zero. Additionally, both Literal [*t*(23) = 2.614, *p* = 0.016] and Metaphor [*t*(25) = 2.228, *p* = 0.035] conditions differed from Abstract sentences, but Literal and Metaphor did not differ from each other [*t*(24) = 0.22].

At the 450 ms SOA, no significant effects emerged, although both Literal and Metaphor sentences were marginally different from zero [Literal: *t*(12) = 1.869, *p* = 0.086; Metaphor: *t*(12) = 2.16, *p* = 0.052]. Notably, the Metaphor sentences were faster following motor cortex TMS at the 450 ms SOA, while all other effects were slower for motor cortex TMS (see Figure 1).

A linear mixed effects model was also performed at each SOA using the lme4 package in R (Bates et al., 2015) included Subject and Item as random effects which were allowed to vary with random intercepts, as well as Site and Condition as dummy coded fixed effects with the Control site and Abstract sentences as control levels, respectively^2^. *p*-values for each contrast were estimated using the lmerTest package (Kuznetsova et al., 2017). The only contrasts that were significant or approached significance were the Literal ^∗^ Site interaction at 300 ms (β = -0.342, *SE* = 0.155, *t* = 1.976, *p* = 0.048); the Metaphor ^∗^ Site interaction at 300 ms (β = -0.293, *SE* = 0.151, *t* = 1.939, *p* = 0.053) and the Literal ^∗^ Site interaction at 450 ms (β = -0.258, *SE* = 0.139, *t* = 1.860, *p* = 0.063).

### Motor-Evoked Potentials

Motor evoked potentials (MEPs) were measured as the maximum peak-to-peak waveform collected from the FDI muscle. Like in the RT analysis, trials were removed if the MEP was greater than 2.5 standard deviations above or below the participant’s mean in a given condition and *z*-scored within-participant.

**FIGURE 3:**
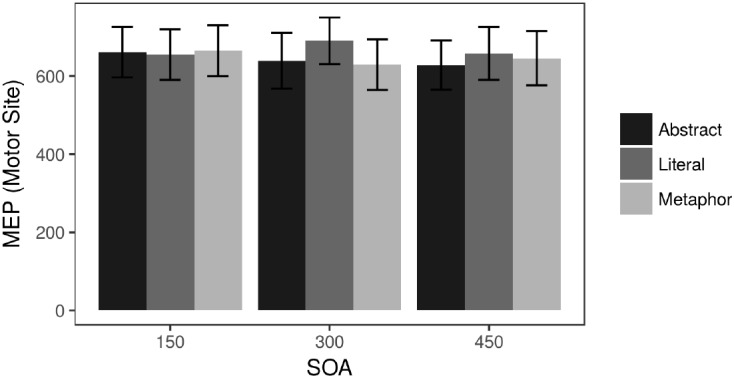
Raw MEP data (microvolts) during Motor site stimulation.

There was a strong effect of motor cortex stimulation on MEPs relative to the Control condition, confirming that the Motor Site was in fact stimulating the FDI [*t*(2048) = 65.2, *p* < 0.0001]. Among the Motor Site data, a within-subject ANOVA did not reveal any main effects nor an interaction. Figure 3 depicts the raw Motor Site MEP results. The MEP results were also analyzed as a difference score between Motor and Control site TMS to examine any general effects of applying TMS, and the pattern of the results was the same as those reported here (that is, MEPs lacked any signal during Control stimulation).

Looking at individual SOAs during Motor Site stimulation, there were no significant differences between conditions at the 150 ms SOA. At 300 ms, Literal sentences elicited significantly higher MEPs than Metaphor sentences [*t*(302) = 2.54, *p* = 0.011] and marginally higher MEPs than the Abstract sentences [*t*(295) = 1.96, *p* = 0.051]. At the 450 ms SOA, the only trending effect was a weak difference between Literal and Abstract sentences [*t*(311) = 1.66, *p* = 0.098]. A linear mixed effects analysis analogous to the RT analysis above showed a Literal ^∗^ Site interaction at 300 ms (β = 51.3, *SE* = 24.9, *t* = 2.059, *p* = 0.047) and no other effects.

## Discussion

The current study used TMS to disrupt the primary hand motor cortex at multiple timepoints during action verb processing, with two goals. First, it assessed whether the motor cortex plays a causal, rather than secondary, role in action verb processing. Second, it assessed the time course of motor cortex involvement in metaphor comprehension. During metaphor comprehension, an effect of TMS suggests that the literal meaning of the verb is (still) being accessed at the timepoint of stimulation. Results showed that motor cortex TMS affected both literal and metaphor sentences at the 300 ms SOA, suggesting a causal role of the primary motor cortex in both types of action verb processing.

Reaction time effects only emerged beginning with the 300 ms SOA, and responses in the abstract condition were not influenced by motor cortex TMS. Comprehension of the literal sentences was slowed following TMS at 300 ms, consistent with a role for the motor cortex in processing literal action verbs. The metaphor effect emerged as significant in the ANOVA but as marginal in the mixed effects analysis, hence this finding would benefit from replication. Note that the effects of TMS are meaningful only in relation to control stimulation, not in terms of their absolute values. Absolute RTs are affected by inherent characteristics of the stimuli (sentences in some conditions may be relatively easier to process), and by non-specific effects of TMS (such as distraction due to sensation on the scalp). A comparison with the control site stimulation minimizes these effects.

Regarding metaphoric sentences, results show that the motor cortex remains involved in comprehension 300 ms after verb onset. This is not consistent with the hypothesis that metaphors are processed as homophones, since in this view access to the incongruent literal meaning should be suppressed and unnecessary by approximately 200 ms. Instead, both literal and metaphor sentences appear to be affected by TMS at the 300 ms SOA. Both interpretations require motor semantics fairly late in processing, supporting a polysemous view of metaphor meaning.

Indirect access theories of metaphor processing suggest that the literal meaning is necessarily accessed first, and deemed to be incompatible, after which the metaphoric meaning is accessed (Searle, 1979; Janus and Bever, 1985). Direct access models suggest that metaphoric meaning can be accessed directly, without the necessity to access literal meaning (Gibbs, 1994; Glucksberg, 2008). The Graded Salience Hypothesis (Giora, 2003) is an intermediate view that suggests that direct access is dependent on the salience and familiarity of the stimuli. The foundational assumption in this debate is that that are two independent, separate meanings: literal and metaphoric. This assumption gives rise to the problem of order of access. Here, we challenge this assumption, at least with respect to predicate metaphors, and suggest that the metaphoric meaning is not independent from the literal meanings, but contains some relevant aspects of it. Thus, when using the metaphor “grasp an idea,” the metaphoric sense (“to understand”) is not an abstract meaning that is directly or indirectly accessed. Instead, it is processed as having some characteristics of a physical grasp.

The results here are compatible with the Underspecification Model of figurative language (Frisson and Pickering, 2001), which proposes that a single underspecified meaning of a word is necessarily activated during sentence reading, and this meaning is gradually “honed” by context. The current results suggest that the putative underspecified meaning has a motor component, and is not abstract.

There are suggestions that processing novel or creative metaphors may require recruitment of extra resources relative to literal actions or overlearned idioms (Arzouan et al., 2007; Desai et al., 2011; Desai et al., 2013; Lai and Curran, 2013). Metaphors used in the study, however, were familiar overall (on average over 5 on a 7 point familiarity scale). This suggests that the causal involvement of the motor cortex does not reflect an extra resource brought in to deal with highly novel stimuli, but rather it is an inherent part of the grounding of metaphoric (and literal) meaning.

These results support what neuroimaging studies have suggested, that the motor cortex plays a role in action verb processing, and complements neuroimaging findings by lending evidence in favor of causality. Some amodal theories raise the notion of conceptual “cores” that are purported to contain some essential aspects of meaning. However, the existence of cores is questionable (Lebois et al., 2015). Even if assumed to exist, it is not clear that cores are necessarily amodal. If a conceptual core contains centrally important or defining features of concepts, then it should include motor features for action verbs and also be sufficient for comprehension under most conditions, including a sentence sensibility judgment task. Disruption of non-core regions should therefore not impact comprehension. The current results (along with a body of literature including behavioral, patient, and imaging studies) suggest, instead, that features located in sensory-motor regions constitute information that is not epiphenomenal or peripheral to the concept.

At the 450 ms SOA, no significant effects of TMS were observed, but some trends emerged that suggested suppression of action meaning for metaphors but not for literals. Beyond the lack of a robust statistical effect, we don’t derive strong interpretations from this result for another reason. To match the Desai et al. (2013) fMRI experiment, the stimuli in the current study were presented in two parts and not using rapid serial visual presentation of single words. Due to this, the entire phrase starting with the verb was available to the participants (e.g., “lifted the pebble from the ground,” and “lifted the nation out of poverty”). Eye-tracking studies suggest that fixation durations average around 225–250 ms (Rayner et al., 2007; Henderson et al., 2013). At the 450 ms timepoint, it is possible and likely that the fixation has shifted from the verb and processing has started on subsequent words. Hence, some contribution from subsequent nouns (such as ‘pebble’ or ‘nation’) may also reflected in the results (as function words such as ‘is’ are often processed parafoveally and frequently skipped). These nouns in the literal sentences tend to be concrete and manipulable (‘pebble’), while those in the metaphoric sentences tend to be abstract (‘nation’). Due to this limitation of the design, we do not draw clear conclusions from the statistically weak results at the 450 ms SOA, but it appears that during the time window when action verbs are being integrated into sentence context, disrupting the motor cortex does not hinder metaphor comprehension and may even aid in it (given the small facilitatory effect of motor cortex TMS).

Results at the 150 ms SOA are non-significant, although there is a qualitative pattern suggesting that literal and metaphor sentences may be weakly influenced by TMS even during the earliest time window. In auditory word processing, it has been suggested that meaning is accessed as early as 80 ms following the disambiguation point in word presentation, that is, after a sufficient portion of the word has been perceived to distinguish it from all other words (Papeo and Caramazza, 2014; Shtyrov et al., 2014). However, visual word processing typically does not have an early disambiguation point. It is possible that priming the motor system (e.g., by requiring a finger tap in advance of action verb processing) can speed up motor cortex involvement in language processing and show effects as early as 150 ms (Mollo et al., 2016). Thus, the precise onset of motor cortex activity during action verb processing may be somewhat flexible, and weak effects may indeed exist in the current experiment in the earliest time window.

We also examined the MEPs elicited during the experiment. During the 300 ms SOA, TMS during literal sentences elicited slightly greater MEPs than the other sentence types. Although significant MEPs were not detected for metaphoric sentences in the current study, the absence of statistical significance should be interpreted carefully. It is possible that similar studies which measure only corticospinal activity and not behavior are omitting a highly sensitive measure of motor cortex activity. In Cacciari et al. (2011), MEPs showed effects of TMS for both literal and metaphor sentences. A key difference between that and the current study is that the former was designed to measure MEPs and hence required no manual response, while the current study was designed to collect behavior during sentence comprehension and hence required a response. The inclusion of manual button press responses of the contralateral hand almost certainly introduced noise into the responses of the current experiment’s MEPs. A second important difference between the studies is that Cacciari et al. (2011) measured MEPs at the final word of the sentence, while we measured response to the verb, before which only the noun phrase provided the context. Thus, null MEP results for metaphors in the current study should not be interpreted strongly, or as necessarily contradicting Cacciari et al. (2011).

While the results of the study lend support to an embodied or a hybrid account, there are several limitations that invite further investigations and replications. These limitations include a relatively small number of sentences (12–14 sentences per conditions per SOA), as well as a limited amount of context (a noun phrase) supporting a literal or metaphoric interpretation of the verb. The small stimulus set was used to minimize participant fatigue and discomfort during stimulation, but also may have limited the statistical strength of the results, exemplified by, for example, the trending results for metaphors in the mixed effects analysis. The two-part (as opposed to RSVP) presentation also limits the interpretation of the 450 ms timepoint. Hence, the current results should be treated with caution. Future studies using a larger stimulus set, more extensive context, and RSVP presentation can provide corroborative evidence. Regarding MEPs, more conclusive results can be provided by studies that omit the manual task. Studies that stimulate other action-related areas, such as the anterior inferior parietal lobule and posterior inferior frontal gyrus, would also be valuable, as the involvement of action circuits in semantics is likely to extend well beyond primary motor cortex, as suggested by imaging and lesion studies.

In summary, results provide preliminary evidence that TMS to the motor cortex affects successful comprehension of both literal and metaphor interpretations of action verbs. This finding suggests a causal role for the primary motor cortex in action verb processing. In addition, the effect of motor cortex TMS on metaphor processing lends preliminary support to a view of figurative language in which metaphors are processed like polysemous meanings, where the abstract meaning is tied to the meanings of their literal interpretations.

## Ethics Statement

This study was carried out in accordance with the recommendations of the Institutional Review Board at the University of South Carolina with written informed consent from all subjects. All subjects gave written informed consent in accordance with the Declaration of Helsinki. The protocol was approved by the Institutional Review Board.

## Author Contributions

RD and MR conceived and designed the study. MR and OH developed the methodology and collected the results. MR analyzed the results and drafted the manuscript. All authors participated in editing the manuscript.

## Conflict of Interest Statement

The authors declare that the research was conducted in the absence of any commercial or financial relationships that could be construed as a potential conflict of interest.

## Footnotes

1 https://www.microsoft.com/cognitive-services/en-us/web-language-model-api

2 The full model for a given dataset (e.g., the RTs at 300 ms) was lmer(data = data300, RT ∼ (1| Item) + (1| Subject) + Site ^∗^ Condition).
